# 574. Daily Antibiotic Utilization Prescription Audit in the Infectious disease (ID) and Non-ID Department in Hospitalized Patients: A Prospective Case-Control study in a Teritiary care hospital

**DOI:** 10.1093/ofid/ofad500.643

**Published:** 2023-11-27

**Authors:** Suresh Kumar Dorairajan, G Pavithra, Nivetha Hari, Jenifer F Angel, Oviya Vijayakumar, Kezia V Eiphan

**Affiliations:** Apollo hospital, Chennai, Tamil Nadu, India; MMM Hospital, chennai, Tamil Nadu, India; MMM Hospital, chennai, Tamil Nadu, India; MMM Hospital, chennai, Tamil Nadu, India; MMM Hospital, chennai, Tamil Nadu, India; MMM Hospital, chennai, Tamil Nadu, India

## Abstract

**Background:**

Antimicrobial prescription audits were often carried out to reduce the overuse of antibiotics. Usually, audits were performed over periodical intervals. Daily antibiotics audits were time consuming and labour intensive. Though it"s practically impossible, we tried. However, we conducted this audit over a three-month period as daily audit and the cases were regularly followed. It is the prospective case-control study of antibiotic utilizations from prescriptions on ID and non-ID department.

**Methods:**

This Prospective Case-Control study was conducted in tertiary care hospital in India for a period of 3months (Jan-mar2023). Data regarding demographics, antibiotic therapy, diagnosis, rational antibiotic usage (indications mentioned or not), type of therapy (surgical prophylaxis, definitive and empirical therapy) and 48hrs review of antibiotic prescriptions (escalation, de-escalation, continuation and dis-continuation therapy) of both groups were collected by Pharm-D interns from the medical records and analysed using SPSS version 26.0.

**Results:**

During the study period, 899 subjects’ medical records were audited for antibiotic usage from the prescriptions. The comparative data were shown in table-1.

**Figure:**
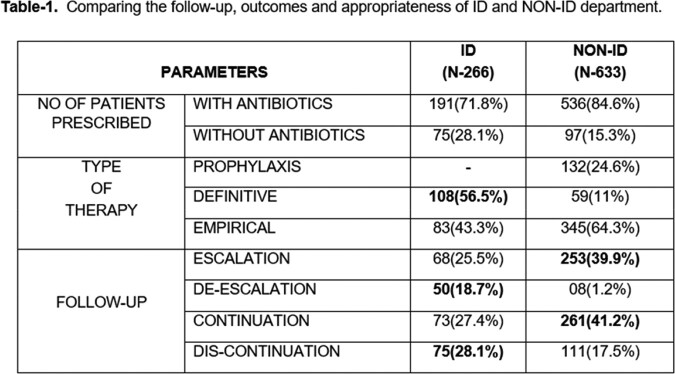

**Conclusion:**

1/3^rd^ of the cases acquired ID opinion which is significantly less than the non-ID department. Predominantly ID opinion is acquired when culture shows positive results. In most cases, the indications were mentioned only by the ID and not by the non-ID team. Discontinuation (28.1%) and de-escalations (18.7%) done by the ID was found to be significantly higher than non-ID. Based on this, we conclude that ID opinion play a crucial role in selection of antibiotics, both in terms of definitive and empirical choices. This study has certain limitations where, outcomes will be followed further.

**Disclosures:**

**All Authors**: No reported disclosures

